# FBXW7 E3 ubiquitin ligase: degrading, not degrading, or being degraded

**DOI:** 10.1007/s13238-019-0652-x

**Published:** 2019-07-24

**Authors:** Huiyin Lan, Yi Sun

**Affiliations:** 1 The Cancer Institute of the Second Affiliated Hospital and Institute of Translational Medicine, Zhejiang University School of Medicine, Hangzhou, 310029 China; 2 Department of Radiation Oncology, Zhejiang Key Lab of Radiation Oncology, Zhejiang Cancer Hospital, Hangzhou, 310022 China; 3 Division of Radiation and Cancer Biology, Department of Radiation Oncology, University of Michigan, Ann Arbor, MI 48109 USA

It is well-established that FBXW7 acts as a tumor suppressor by promoting the polyubiquitylation via the K48 linkage of a broad range of oncoproteins for proteasome degradation. We and the others found that FBXW7 also recognizes non-canonical substrates, such as XRCC4 or γ-catenin for polyubiquitylation via the K63 linkage, not for degradation, but for functional modulation. Most recently, we found that FBXW7 binds to a pseudo-substrate LSD1, not for degradation, rather being self-degraded. Thus, FBXW7 has three modes of action in a manner dependent of substrates, leading to different biological consequences.

FBXW7 is an F-box protein, acting as substrate recognition subunit of SCF (SKP1-CUL1-F-box protein) E3 ubiquitin ligase complex. It has been well-established that FBXW7 functions as a typical tumor suppressor by targeting a large number of critical human oncoproteins for ubiquitylation and proteasome degradation. These oncoprotein substrates include cyclin E, c-JUN, c-MYC, NOTCH-1, and MCL-1, among others (Welcker and Clurman, 2008), and most of these substrates are transcription factors or key signaling molecules that regulate a wide range of cellular process, leading to proliferation and tumor progression. It is, therefore, not surprising that loss-of-function of FBXW7 by genomic deletion or mutation, promoter hypermethylation frequently occurs in various human cancers (Welcker and Clurman, 2008; Nakayama and Nakayama, 2006; Wang et al., 2014). Specifically, cholangiocarcinoma and T cell acute lymphoblastic leukaemias (T-ALL) harbor the highest mutation rates of FBXW7 up to approximately 30%; Pancreatic, gastric, colon carcinomas, prostate and endometrial cancers had mutation frequencies in the range 4%–15% (Welcker and Clurman, 2008). And it has been shown that tissue specific ablation of Fbxw7 accelerated tumorigenesis in various mouse models (Mao et al., 2004; Zhou et al., 2013).

In response to a degradation signal, the substrates of FBXW7 are often phosphorylated by a kinase on the threonine or serine residue within an evolutionarily conserved motif, designated as CPD (Cdc4 phosphodegron), followed by FBXW7 binding and subsequent polyubiquitylation by SCF E3 ligase (Wang et al., 2014). The ubiquitin contains 7 lysine residues (K6, K11, K27, K29, K33, K48 and K63), and ubiquitin chains assembled to targeted substrates could be diversely formed through its different linkage on a particular lysine residue or even formed between the amino group of methionine residues of ubiquitin and the carboxy group of glycine residues of another (termed as K0/linear ubiquitin linkage) (Kirisako et al., 2006), leading to different chains of topology that determines the fate of the substrates (Ikeda and Dikic, 2008). Among all ubiquitin chains, the linkage via K48 and K63 is well-studied and defined. The polyubiquitylated substrates via the K48 linkage are readily recognized by proteasome for targeted degradation, whereas those with the K63 linkage are not doomed for degradation, rather for altered functions, such as setting up a platform to facilitate the recruitment of other proteins in the formation of a functional complex (Ikeda and Dikic, 2008).

**Figure 1:**
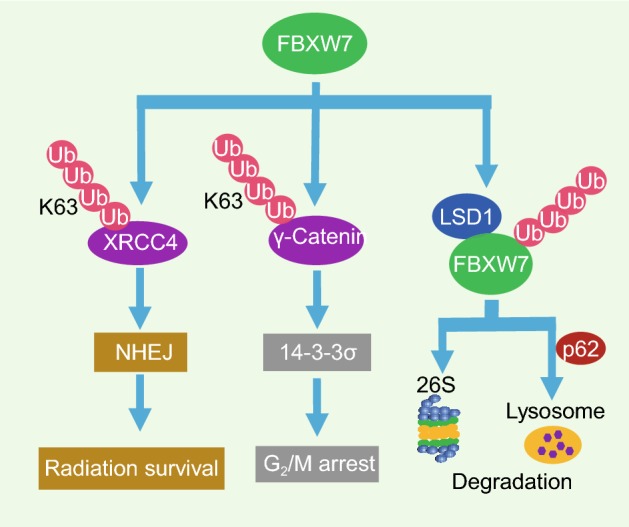
**Non-canonical substrates of FBXW7**. 1) FBXW7 binds to XRCC4 and promotes its polyubiquitylation via the K63 linkage, not for degradation, but for facilitating Ku70/80 recruitment to enhance the NHEJ repair; 2) FBXW7 promotes γ-catenin polyubiquitylation via the K63 linkage to increase the transcriptional expression of 14-3-3σ, and to enhance the activity in growth suppression and G2/M arrest; 3) LSD1, a pseudo-substrate of FBXW7, destabilizes FBXW7 by disrupting its dimerization and triggering its self-ubiquitylation for degradation via both proteasome and lysosome pathways

Until recent, most known FBXW7 substrates are polyubiquitylated via the K48 linkage for proteasome degradation. Two exceptions were reported from our laboratory and another laboratory, showing that FBXW7 also recognizes non-canonical substrates for polyubiquitylation via the K63 linkage (Zhang et al., 2016; Li et al., 2018). Most recently, we found that FBXW7 binds to a pseudo substrate, LSD1 (lysine-specific demethylase 1) with a surprising consequence: not promoting LSD1 ubiquitylation, rather being self-ubiquitylated and degraded (Lan et al., 2019). Here, we summarize the effect of FBXW7 on these non-degradative substrates and the functional consequence to the substrates and FBXW7 itself:

We recently found that FBXW7 enhances nonhomologous end-joining (NHEJ) repair by targeting XRCC4 for polyubiquitylation via the K63 linkage (Zhang et al., 2016) (Fig. 1). Mechanistic studies revealed that, upon radiation, FBXW7 is phosphorylated at the S26 by ATM, and recruited to DNA damage sites. Radiation also activates DNA-PK to phosphorylate XRCC4, a core protein involving NHEJ. FBXW7 meets and binds phosphorylated XRCC4 at the damage sites on its CPD motif to promote XRCC4 polyubiquitylation at the K296 via the K63 linkage, not for degradation, but facilitating recruitment of Ku70/80 heterodimer to enhance efficiency of NHEJ repair. Moreover, we demonstrated that UBC13, an E2 that predominantly mediates K63-linked polyubiquitylation (VanDemark et al., 2001), was involved in the FBXW7-XRCC4 interaction, and UBC13 knockdown inhibited the NHEJ repair, which further support the notion that FBXW7 favors the K63 linkage towards XRCC4. To further elucidate the importance of FBXW7-XRCC4 interaction in NHEJ repair, several mutants, including the ubiquitin-resistant XRCC4-K296R, the recruitment-deficient FBXW7-S26A, and the phosphorylation-resistant XRCC4-S325A/S326A were generated, and all mutants showed significantly reduced NHEJ repair capacity. Biologically, these FBXW7 mutants are less effective in protecting cells from radiation-induced damage, as measured by clonogenic survival assay. Consistently, FBXW7 inactivation via genetic or pharmacological approach inhibits the NHEJ repair and sensitizes cancer cells to radiation (Zhang et al., 2016).

Collectively, we defined that XRCC4 is the first non-degradative FBXW7 substrate. FBXW7-mediated K63-linked polyubiquitylation of XRCC4 facilitates the NHEJ repair and confers radiation protection. The translation implication of this study is that cancer patients with inactivated FBXW7 might have a better prognosis with radiotherapy.

In last year, another group showed that FBXW7 promotes K63-linked polyubiquitylation of γ-catenin to suppress cell proliferation and G_2_/M cell cycle transition (Li et al., 2018) (Fig. 1). In that report, the authors found that γ-catenin was a novel binding partner of FBXW7, whereas the stability of γ-catenin was not changed by FBXW7 manipulation. Using three ubiquitin mutants including K11, K48 and K63, FBXW7 was found to specifically promote K63-linked polyubiquitylation of γ-catenin. The functional consequence of γ-catenin polyubiquitylation was demonstrated by an enhanced activity in transcription of γ-catenin downstream gene 14-3-3σ in a manner dependent of FBXW7. Biologically, FBXW7 enhanced the function of γ-catenin in its capacity of suppressing cell proliferation and inducing G_2_/M arrest (Li et al., 2018).

This study, therefore, uncovered γ-catenin as a new non-degradative substrate subject to FBXW7-mediated polyubiquitylation via the K63 linkage for enhanced function, thus providing a new mechanism of FBXW7 in negative regulation of cell cycle progression and cell proliferation.

Most recently, we identified LSD1 (also known as KDM1A) as a pseudo-substrate of FBXW7, which was neither for targeted degradation, nor for altered function, rather unexpectedly triggering FBXW7 self-ubiquitylation and subsequent degradation (Lan et al., 2019) (Fig. 1). LSD1 is the first histone demethylase identified as a transcriptional repressor to regulate gene expression via catalyzing the demethylation of mono- and di-methylated histone 3 lysine 4 (H3K4Me1, H3K4Me2) (Shi et al., 2004). Using co-immunoprecipitation, we found that FBXW7 directly binds to LSD1 via the CPD binding sites, but fails to promote LSD1 ubiquitylation nor the protein stability. Surprisingly, FBXW7-LSD1 binding significantly attenuated FBXW7 binding with its substrates, such as c-MYC, cyclin E and NOTCH 1 (Welcker and Clurman, 2008), suggesting that the pseudo-substrate could compete with *bona fide* substrates for FBXW7 binding. Furthermore, we found that LSD1 negatively regulates the protein level and half-life of FBXW7 in a manner dependent of its FBXW7 binding, but independent of its demethylase activity. Interestingly, LSD1-induced FBXW7 degradation can only be completely rescued by the combination of inhibitors targeting both proteasome (e.g., MG132) and lysosome (e.g., CQ, chloroquine), implying that autophagy also plays a role in FBXW7 degradation. Mechanistic study revealed that LSD1 disrupts FBXW7 dimerization and accelerates monomeric FBXW7 for self-ubiquitylation, which is then degraded via both proteasome and p62-mediated lysosomal pathways. Biological, we found that LSD1 abrogates FBXW7 functions in growth suppression, NHEJ repair and radiation protection (Lan et al., 2019).

Collectively, we identified LSD1 as a FBXW7 pseudo-substrate, which is not being ubiquitylated or degraded, but triggers FBXW7 self-ubiquitylation and degradation. We, therefore, established a novel demethylase-independent oncogenic mechanism of LSD1 via targeting FBXW7. Our study provided a novel strategy to reactivate FBXW7 in human cancer with LSD1 overexpression via targeting LSD1 protein for degradation, rather than merely inhibiting its enzymatic activity (Lan et al., 2019).

Taken together, the studies described here reveal that FBXW7 not only targets a number of oncogenic proteins for degradation, but also regulate protein functions via K63-linked polyubiquitylation. Furthermore, FBXW7 itself is subjected to negative regulation by its pseudo-substrate, LSD1, leading to its inactivation (Fig. 1). The identification and characterization of these non-degradative substrates largely extends the mechanism of FBXW7 action as a tumor suppressor.
